# Randomized quantile residuals for diagnosing zero-inflated generalized linear mixed models with applications to microbiome count data

**DOI:** 10.1186/s12859-021-04371-6

**Published:** 2021-11-25

**Authors:** Wei Bai, Mei Dong, Longhai Li, Cindy Feng, Wei Xu

**Affiliations:** 1 Department of Mathematics and Statistics, University of Saskatchewan, Saskatoon, CA Canada; 2 Dalla Lana School of Public Health, University of Toronto, Toronto, CA Canada; 3 Department of Community Health and Epidemiology, Dalhousie University, Halifax, CA Canada

## Abstract

**Background:**

For differential abundance analysis, zero-inflated generalized linear models, typically zero-inflated NB models, have been increasingly used to model microbiome and other sequencing count data. A common assumption in estimating the false discovery rate is that the *p* values are uniformly distributed under the null hypothesis, which demands that the postulated model fit the count data adequately. Mis-specification of the distribution of the count data may lead to excess false discoveries. Therefore, model checking is critical to control the FDR at a nominal level in differential abundance analysis. Increasing studies show that the method of randomized quantile residual (RQR) performs well in diagnosing count regression models. However, the performance of RQR in diagnosing zero-inflated GLMMs for sequencing count data has not been extensively investigated in the literature.

**Results:**

We conduct large-scale simulation studies to investigate the performance of the RQRs for zero-inflated GLMMs. The simulation studies show that the type I error rates of the GOF tests with RQRs are very close to the nominal level; in addition, the scatter-plots and Q–Q plots of RQRs are useful in discerning the good and bad models. We also apply the RQRs to diagnose six GLMMs to a real microbiome dataset. The results show that the OTU counts at the genus level of this dataset (after a truncation treatment) can be modelled well by zero-inflated and zero-modified NB models.

**Conclusion:**

RQR is an excellent tool for diagnosing GLMMs for zero-inflated count data, particularly the sequencing count data arising in microbiome studies. In the supplementary materials, we provided two generic R functions, called rqr.glmmtmb and rqr.hurdle.glmmtmb, for calculating the RQRs given fitting outputs of the R package glmmTMB.

**Supplementary Information:**

The online version contains supplementary material available at 10.1186/s12859-021-04371-6.

## Introduction

The next-generation sequencing technologies, such as RNA and microbiome sequencing, typically produce count data measuring the abundance of a large set of nucleic acid sequences. A central goal of analyzing sequencing count data is to identify the sequences with differential abundance under different conditions. For example, many human microbiome studies aim to identify microbial taxa with differential abundance in healthy and diseased patients [1]. In microbiome data, the microbial taxa are represented by nucleic acid sequences called operational taxonomic units (OTUs) at different levels in an independent taxonomic way [2, 3]. In this paper, we use microbiome sequencing data as an example in our discussion for the simplicity of terminologies. However, the applicability of the methods discussed in this paper is not limited to microbiome count data.

Generalized linear models (GLM) are commonly used to model the sequencing count data. Negative-binomial (NB) based regression models are used in many widely used bioinformatics analysis tools and methods [4–8]. Excessive zeros are commonly observed in sequencing count data. For microbiome data, the reason for the excessive zeros is either due to the absence of taxa (structural zeros) or the presence of taxa with a low frequency, which results in observed counts below detection limits (sampling zeros). One way to deal with excessive zeros is to use a zero-inflated model [9], which is a mixture of a regular count regression model, such as Poisson or NB model, and logistic regression to model the excessive zeros. Another way is to use a zero-modified model, also called a hurdle model [10], with one part being a logistic regression model to model the zeros and the other part being the truncated count regression model (e.g. truncated NB) to model the positive count data. Moreover, subjects in microbiome data often have clustering structures, for example, humans from the same family or plants from the same plot. To model the association of the abundance of taxa with such environmental factors, we often use random effects to account for the clustering structure in microbiome study[8, 11]. Increasing evidence [12] shows that the zero-inflated models can give better fits (measured by AIC) to sequencing count data than the corresponding models without a zero-inflation component. As such, recently zero-inflated generalized linear models with or without random effects, typically zero-inflated NB (ZINB) models, have been increasingly used to model microbiome and other sequencing count data [1, 11–22]. In addition to the applications in sequencing count data, zero-inflated generalized linear mixed models (GLMM) have also been widely applied to model count data arising in a wide variety of fields, such as ecology and epidemiology [23–29]. The aforementioned features, including zero-inflation, over-dispersion, and clustering, are also commonly observed in the count data collected from these areas.

A common drawback of using a parametric model such as a ZINB model is that the model may fail to provide an adequate fit to a dataset. For example, Hawinkel et al. [30] proposed a specific smooth test for checking the GOF of NB models with applications to a large set of sequencing count datasets and concluded that NB models do not fit well to many of the sequence datasets. The model mis-specification problem has been largely neglected in today’s statistical modelling practice, including in bioinformatics. However, the conclusions drawn from poorly fit models may be seriously misleading. In differential abundance analysis, the *p* values for all taxa are typically converted into q-values or passed to an FDR controlling procedure [31] for controlling the false discovery rate (FDR) at a nominal level. A common assumption in estimating FDR is that the *p* values are uniformly distributed under the null hypothesis, which holds when the postulated model fits the data of all taxa adequately. When the model is mis-specified, the distribution of the *p* values under the null hypothesis may be far away from the uniform distribution, often resulting in an underestimate of the true FDR and excess false discoveries. The excess false discoveries may then lead to costly but fruitless follow-up studies on the falsely identified taxa. This problem has been discussed in detail and demonstrated with simulation studies by [17, 32, 33]. As such, model checking is critical to control the FDR at a nominal level in differential abundance analysis.

It is challenging to conduct model checking and diagnostics for generalized linear models for count data. Akaike’s information criterion (AIC) is commonly used to compare the goodness-of-fits of competing models. However, AIC cannot check whether a postulated model is close enough to the true model (e.g. the adequacy of a model). Examining the normality of Pearson’s residuals is a standard tool for diagnosing normal regression. Pearson and deviance residuals are often used to diagnose generalized linear models. However, both Pearson and deviance residuals are far from normality for count regression. In particular, Pearson and deviance residuals cluster on curves due to the discreteness [34, 35]. Due to the lack of normality, it is challenging to conduct model checking and diagnostics with Pearson and deviance residuals for count regression. Recently, a few GOF tests based on the cumulative sums of residuals [36, 37] have been developed for the zero-inflated models [38]. However, these GOF tests cannot be used to reveal the nature of model discrepancy for suggesting certain strategies to improve a poorly fit model. The smooth test proposed in [30] is difficult to be extended to more flexible models, such as zero-inflated models. In addition, the smooth test is a likelihood ratio test (LRT). The validity of the *p* value of an LRT test itself depends on the correctness of the assumed null model.

The method of randomized quantile residual (RQR) was proposed by Dunn and Smyth [39] to overcome the challenges of diagnosing count regression. The central idea of the RQR is to randomize the predictive *p* value (i.e. tail probability of CDF for response) into a uniform random number. With this randomization, the distribution of RQRs is a standard normal under the true model for the dataset. Therefore, we can conduct model diagnostics with RQRs for count regression models in the same way for normal regression. Recently, Feng et al. [35] compared the performance of conducting GOFs with RQRs in generalized linear models and concluded that the GOF tests with RQRs are well-calibrated and have good power. The method of RQR has also been increasingly applied to some zero-inflated regression models without considering random effects [40–44]. However, to the best of our knowledge, the method of RQR has not been applied to sequencing count data. Furthermore, the performance of RQRs in diagnosing zero-inflated GLMMs for sequencing count data has not been extensively investigated in the literature.

The primary objective of this article is to demonstrate that the method of RQR performs very well for diagnosing zero-inflated GLMMs and is particularly suitable for checking whether such models provide adequate fits to sequencing count data. The rest of the article is organized as follows. Sections “Generalized linear mixed models for zero-inflated data” and “Randomized quantile residuals” describes zero-inflated GLMMs and the method of RQRs respectively. In Section “Simulation studies” we report the results of performing large-scale simulation studies to investigate the performance of RQRs for zero-inflated GLMMs. The simulation studies show that the probabilities of type I errors of the GOF tests with RQRs are very close to the nominal level, and the GOF tests have excellent power; in addition, the scatter-plots and Q–Q plots of RQRs are useful in discerning the good and bad models. In Section “Application to a real human microbiome dataset” we apply the RQR to diagnose six GLMMs to a real microbiome dataset. The results show that the OTU counts at the genus level of this dataset (after a truncation treatment) can be modelled well by zero-inflated and zero-modified NB models.

## Generalized linear mixed models for zero-inflated data

In this section, we will describe two commonly used models, zero-inflated and zero-modified, for handling excessive zeros. Zero-modified models refer to hurdle models that are often used in the literature.

### Zero-inflated mixed-effects models

A zero-inflated model is a mixture of two distributions. One part is a binary distribution describing $y_i$ being zero or not. The second part is a count regression model, such as Poisson distribution or NB regression distribution. The zeros that we observe from a dataset are then a mixture of zeros from these two distributions, referred to as structural zeros and sampling zeros, respectively. The PMF and CDF of a zero-inflated model can be written as follows:

1 $$\begin{aligned} f(y_i)= & {} \left\{ \begin{array}{ll} p_i + \left( 1- p_i\right) g\left( y_i\right) , &{}\quad \text{ for } y_i = 0 \\ \left( 1 - p_i\right) g\left( y_i\right) , &{}\quad \text{ for } y_i >0 \end{array} \right. \end{aligned}$$

2 $$\begin{aligned} F\left( y_i=J\right)= & {} \sum _{j=0}^{J}f\left( y_i=j\right) = p_i F_0(J) + \left( 1-p_i\right) G(J) \end{aligned}$$

where $F_0(\cdot )$ is the CDF of point mass 0, ie., $F_0(J) = 0 \text{ if } J < 0; = 1 \text{ otherwise }$; $g(\cdot )$ is the PMF of a distribution for counts (including 0); $G(\cdot )$ is the CDF of $g(\cdot )$; $p_i$ is the mixture proportion. In particular, in a ZINB model, the NB distribution is used to model the counts with $g(\cdot )$ given as follows:

3 $$\begin{aligned} g(y_i)= & {} f{}^{\text{ NB }}\left( y_i; \mu _{i},\theta \right) = \frac{\Gamma \left( y_i+ \theta \right) }{\Gamma (\theta )\Gamma \left( y_i+1\right) } \left( \frac{\theta }{\theta + \mu _i}\right) ^{\theta } \left( \frac{ \mu _i}{\theta +\mu _i}\right) ^{y_i}, \end{aligned}$$

When we use Poisson distribution to describe the counts, the PMF of *g* is given by

4 $$\begin{aligned} g\left( y_i\right) =f{}^{\text{ Pois }}\left( y_i; \mu _{i}\right) = \frac{e^{-\mu _i}\mu _i^{y_i}}{{y_i}!} \end{aligned}$$

The $\mu _i$ in (3) and (4) is the mean of $y_i$, and $\theta >0$ is the inverse dispersion parameter for NB. The NB distribution has heavier tails than the Poisson distribution. When $\theta \rightarrow \infty$, the NB distribution converges to Poisson distribution. The $\mu _i$ and $p_i$ are often linked to fixed and random factors as following:

5 $$\begin{aligned} \begin{aligned} \text{ log }\left( \mu _i\right)&= \text{ offset}_i + X_i\beta + Z_iu\\ \text{ logit }\left( p_i\right)&=\text{ log }\left( \frac{p_i}{1-p_i}\right) = \tilde{X}_i\tilde{\beta }+ \tilde{Z}_i\tilde{u}, \end{aligned} \end{aligned}$$

where $X_i$ and $\tilde{X}_i$ are fixed factors for modelling $\mu _i$ and $p_i$ respectively, which may or may not be identical; $\beta = ( \beta _1 , \beta _2 ,\ldots , \beta _p )^T$ and $\tilde{\beta }= (\tilde{\beta }_1, \tilde{\beta }_2 ,\ldots , \tilde{\beta }_p )^T$ are the corresponding p-dimensional vectors of unknown regression coefficients; $Z_i$ and $\tilde{Z}_i$ are the p-dimensional vectors for the random effects of the conditional count and logistic components of the model, respectively; $u = ( {u}_1 , {u}_2 ,\ldots , {u}_q )^T$ and $\tilde{u} = ( {\tilde{u}}_1 , {\tilde{u}}_2 ,\ldots , {\tilde{u}}_q )^T$ are the unobserved random effects vectors, which are often assumed to be normally distributed $u \sim N(0,\Sigma )$, where $\Sigma$ is a positive definite variance-covariance matrix.

### Zero-modified (hurdle) mixed-effects models

Zero-modified models are also called hurdle models [10]. Both zero-modified and zero-inflated models can be used to model excess zeros in the response variable. In contrast to zero-inflated models, zero-modified models treat zero-count and non-zero outcomes as two completely separate categories, rather than treating the zero-count outcomes as a mixture of structural and sampling zeros. A zero-modified model is composed of two components. A component is a probability distribution for describing the probability of observing value zero or not. The other component models the positive count data using a truncated negative binomial or truncated Poisson, by removing the zero part from the Poisson or NB distribution, and the denominator is to re-normalize the probability so that it still sums to 1. In particular, the PMF and CDF for $y_i$ of a zero-modified model are then written as follows:

6 $$\begin{aligned} f(y_i)= & {} \left\{ \begin{array}{ll} \pi _i, &{}\quad \text{ for } y_i = 0 \\ \left( 1 - \pi _i\right) \frac{g\left( y_i\right) }{1-g(0)}, &{}\quad \text{ for } y_i >0 \end{array} \right. \end{aligned}$$

7 $$\begin{aligned} F\left( y_i=J\right)= & {} \pi _i F_0(J) +\left( 1-\pi _i\right) \frac{G(J)-g(0)}{1-g(0)}I(J>0), \end{aligned}$$

where $I(\cdot )$ is the indicator function, which is equal to 1 when the condition in bracket is true and equal to 0 otherwise.

Similar to zero-inflated models, one can choose different model, $g(\cdot )$, for modelling counts. In ZMNB models, $g(y_i)=f{}^{\text{ NB }}(y_i; \mu _{i},\theta )$; in ZMP models, $g(y_i)=f{}^{\text{ Pois }}(y_i; \mu _{i})$ for ZMP model. The $\mu _i$ and $\pi _i$ are similarly linked to covariates using Eq. (5).

### Comparison of zero-inflated and zero-modified models

When the same $g(\cdot )$ is chosen, the conditional distributions for $y_i$ given $y_i>0$ in the zero-inflated and zero-modified model are identical—both described with the PMF $g(y_i)/(1-g(0))$. The difference of these two models lies in the modelling of $P(y_i=0)$. In zero-modified models, $P(y_i=0)=\pi _i$ is linked to covariates directly. In contrast, in zero-inflated models, $P(y_i=0)=p_i+(1-p_i)g(0)$ is not linked to covariates directly; instead, the mixture proportion $p_i$ is linked to covariates. However, we see that when *g*(0) is very small, which occurs when $\mu _i$ is large, these two models are very close.

## Randomized quantile residuals

Examining the residuals of a regression model is a standard tool for assessing normal regression [45]. Pearson residuals is the raw residual divided by the square root of the variance, written as $r_i = \frac{y_i-\hat{\mu }_i}{\sqrt{ \widehat{V}{(y_i)}}}$, where $\hat{\mu }_i$ is the fitted value and $\widehat{V}{(y_i)}$ is the estimated variance of $y_i$ respectively. Deviance residuals are often defined for generalized linear models [46]. Deviance residual for $y_i$ is defined as the part attributed to $y_i$ in the deviance, which is the difference of log-likelihood of the fitted model to that of a saturated model. For zero-inflated and zero-modified models, it is challenging to find a reasonable saturated model. Most importantly, the distributions of Pearson and deviance residuals are not normal for count regression [35, 39] under the true model. Therefore, the graphical examination of Pearson and deviance residuals are often not informative for diagnosing count regression models. Quantitative assessment of the overall GOF with Pearson and deviance residuals are often based on $\chi ^{2}$ approximation for their sampling distributions. The *Pearson* $\chi ^{2}$ *statistic* is written as, $X^2=\sum _{i=1}^n r_{i}^{2}$, and the *deviance* $(\chi ^{2}$ *statistic)* is written as, $D=\sum _{i=1}^n d_{i}^{2}$. The asymptotic distribution of *D* and $X^2$ under the true model is often assumed to be $\chi ^2_{n-p}$, where *n* is the sample size and *p* is the number of parameters. However, the use of this asymptotic distribution for both $X^{2}$ and *D* lacks theoretical underpinning.

The method of randomized quantile residual (RQR) [39] was proposed to overcome the difficulties of using traditional residuals for diagnosing regression models for discrete outcomes. The idea of RQR is to transform the tail probability of each response value into the equivalent standard normal quantile. Let $F(y_{i}; \mu _{i},\phi )$ denote the cumulative distribution function (CDF) for random variable $y_{i}$, which is parametrized by $\mu _{i}$ (covariate related) and $\phi$ (covariate unrelated, such as size parameter $\theta$ of NB distribution). If the CDF is continuous, $F(y_i;\mu _i,\phi )$ is uniformly distributed on (0, 1) RQRs can then be defined as $q_i = \Phi ^{-1} \{F(y_i;\hat{\mu _i},\hat{\phi }) \},$ where $\Phi ^{-1} ()$ is the quantile function of a standard normal distribution. If the CDF is discrete, randomization is added to make it continuous. To be more specific, let $p(y_i;\mu _i,\phi )$ denote the PMF of $y_{i}$. The randomized tail probability can be defined as:

8 $$\begin{aligned} F^{*}\left( y_i;\mu _i,\phi ,u_i\right) = {\left\{ \begin{array}{ll} F\left( y_i;\mu _i,\phi \right) , &{} \quad F \text { is continuous at }y_{i} \\ F\left( y_i-;\mu _i,\phi \right) +u_i \,p\left( y_i;\mu _i,\phi \right) ,&{} \quad F \text { is discrete at }y_{i} \end{array}\right. } \end{aligned}$$

where $u_i$ is a uniform random variable on $\left[ 0,1 \right]$, and $F(Y_i-;\mu _i,\phi )$ is the lower limit of *F* at  $y_i$. When *F* is discrete, we let $a_i = \lim _{y \rightarrow y_i-}F(y_i; \mu _i, \phi )$ and $b_i = F(y_i; \mu _i, \phi )$, then the RQR for $y_{i}$ is calculated as

9 $$\begin{aligned} q_i=\Phi ^{-1} \left( F^{*}\left( y_i;\mu _i,\phi ,u_i\right) \right) . \end{aligned}$$

Feng et al. [35] gives a detailed explanation of the RQR and illustrates the RQR using a simple GLM model with nonlinear effects.

From the definition (8), the computation of RQRs is straightforward once we can compute the CDF of $y_{i}$. For zero-inflated and zero-modified models, these CDFs are given by Eqs. (2) and (7). We generated two R functions, i.e, rqr.glmmtmb and rqr.hurdle.glmmtmb to calculate RQRs for diagnosing different types of mixed effects counts models. The function rqr.glmmtmb is designed for diagnosing Poisson, NB, ZIP and ZINB mixed-effects models and the function rqr.hurdle.glmmtmb is designed for diagnosing ZMP and ZMNB mixed-effects models. Both functions only request input the fitting results from a model fitted in the glmmTMB package [47], and then these two functions output the RQRs for the corresponding model. These functions are provided in the Additional file 1.

Under the true model with the true parameters, the distribution of RQRs is a standard normal. Based on this null distribution, we could conduct residual diagnostics for count regression models, including zero-inflated GLMMs, in the same way for normal regression models with Pearson’s residuals, including overall GOF tests and graphical examinations such as residual plots and Q–Q plots. However, the standard normality holds only when the true model with the true parameters is used in Eq. (8). The actual performance of the RQR in particular models *with parameters estimated with finite samples* still demands empirical investigation. Feng et al. [35] show that the performance of the RQR is good for generalized linear models. In this paper, we investigate the performance of the RQR in zero-inflated GLMMs with simulated datasets that look like actual microbiome count data.

## Simulation studies

In this section, we present simulation studies to evaluate the performance of RQRs. We simulate data from the ZINB, ZMB, ZIP, and ZMP model, respectively, with varying degrees of excess zeros and over-dispersion. For illustrative purposes, we first assess the GOFs of the true model in comparison with the misspecified models using RQRs and Pearson residuals graphically for a single simulated dataset. Then we simulate 3000 replicate samples to assess the performance of the overall GOF test by testing the normality of the RQRs. The histogram of normality test *p* values and the probability of rejecting the wrong model are presented for comparing the performance of RQRs and Pearson residuals. Section “Description of data generating process” describes data generating process. Section “Simulation results” presents the results of the simulation studies. Section “Illustration of model diagnostics with RQRs for a single dataset” illustrates the performance of RQRs based on a single simulated dataset, and Section “Results of GOF tests based on RQRs with multiple simulated datasets ”presents the results for simulated studies based on replicated datasets.

### Description of data generating process

#### Data generation

We first simulate dataset from zero-inflated model with the outcome variable $Y_{i}$, $i = 1,\ldots , n$, generated as follows,

1. Generate a binary variable $H_{i}$ indicating whether $Y_{i}$ is a structural zero or not, with the probability of $p(H_{i}=0)=p_i$, which is linked to the fixed and random factors using a logistic link function:
  10 $$\begin{aligned} \begin{aligned} \text{ log }\left( \frac{p_i}{1-p_i}\right) = \tilde{\beta }_0+ \sum _{m=1}^{s}\tilde{\beta }_{X_i^{(m)}} + \sum _{n=1}^{t} \tilde{u}_{z_i^{(n)}}, \end{aligned} \end{aligned}$$
  where $\tilde{\beta }_{{X_i}^{(m)}}$ denotes the coefficient associated with the *m*th fixed factor, $m=1, \ldots , s$ and $\tilde{u}_{{Z_i}^{(t)}}$, $t=1, \ldots , T$, denotes the coefficient for the *t*th random effect term.
2. If $H_{i}$ = 0, $Y_{i}=0$; otherwise, $Y_{i}$ is generated from a NB or Poisson model with mean $\mu _{i}$, which is linked to the fixed and random effect terms as follows:
  11 $$\begin{aligned} \log \left( \mu _{i}\right) = \log (T_i) + \beta _{0} + \sum _{m=1}^{s}\beta _{{X_i}^{(m)}}+\sum _{n=1}^{t}u_{{Z_i}^{(n)}}, \end{aligned}$$
  where $\beta _{{X_i}^{(m)}}$ represents the coefficient associated with the *m*th fixed-effect factor, $m=1, \ldots , s$, and $u_{{Z_i}^{(t)}}$ denotes the coefficient associated with the *t*th random factor. $\text{ log }(T_i)$ denotes the offset term to adjust for the varying total sequence reads across the samples.

We also generate data from a zero-modified model with a data generation process similar to one for the zero-inflated model described above. The only difference is that when $H_i>0$, $Y_i$ is generated from a truncated Poisson or NB model. In the zero-modified model, $\mu _i$ represents the mean and $\pi _i$ represents the proportion of zeros.

#### Parameter settings

We generate datasets with $s=3$ fixed factors and $t=2$ random factors and different sample size $n = 50,100,200,400$. Each fixed factor has three levels, and each random factor has two levels. The regression coefficients for the fixed-effects covariates $\beta _i$ follow a normal distribution with mean $\mu =0$, and standard deviation $\sigma =0.1$, and the coefficients for the random effects $u_i$ follow a normal distribution with mean $\mu =0$, and standard deviation $\sigma =2$. The total read $T_i$ follows a Poisson distribution with a mean $\mu =300{,}000$. The shape parameter $\theta$ follows a $\mbox{unif}(2,3)$ distribution.

To investigate the robustness of the performance of RQRs, we consider four scenarios by varying $\tilde{\beta }_0$ and $\beta _0$, in Eqs. (10) and (11), which control the zero proportions (ZP) and dispersion of count component, respectively. More specifically, in scenarios 1 and 2, $\tilde{\beta }_0~=3.5$, which represents the high ZP, while in scenarios 3 and 4, $\tilde{\beta }_0~=-5.5$, which represents the low ZP. In scenarios 1 and 3, $\beta _0=-5.5$ for NB model and $\beta _0=-5.7$ for Poisson model, which represents the relatively high count data, while in scenarios 2 and 4, $\beta _0=-7.8$ for NB model and $\beta _0=-8$ for Poisson model, representing the relatively low count data.

### Simulation results

#### Illustration of model diagnostics with RQRs for a single dataset

**Fig. 1:**
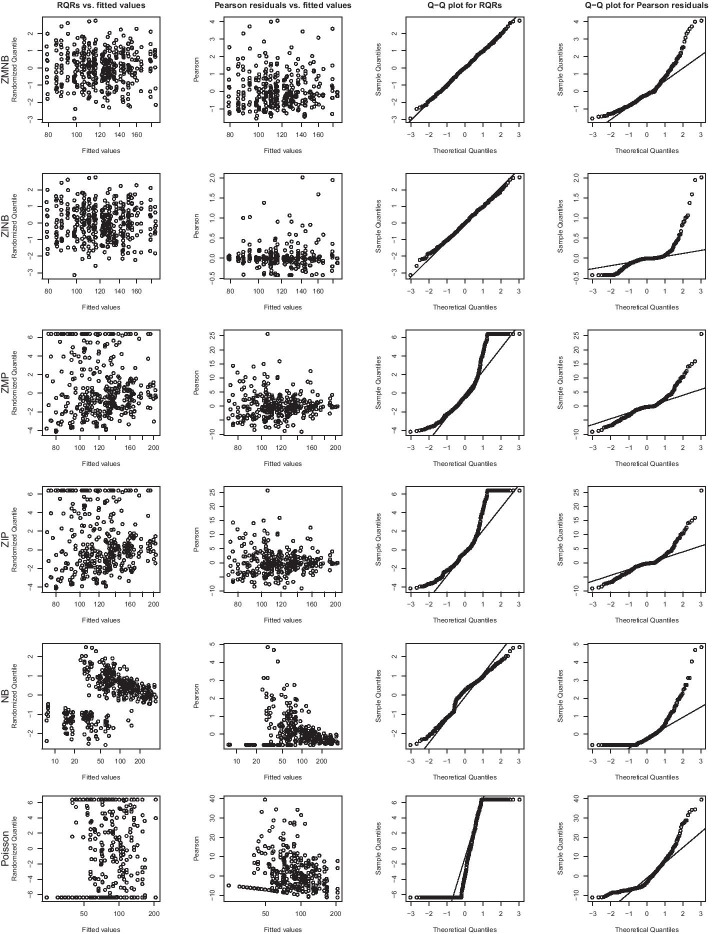
Model diagnostics for a single dataset of $n=400$ samples simulated from a ZMNB model in scenario 4 with parameter settings as low zero proportion and low count. The panels in the first column are the scatter plots of the RQRs vs. fitted values. The panels in the second column depict the scatter plots of the Pearson residuals vs. fitted values. The panels in the third column present the Q–Q plots for RQRs. The panels in the fourth column present the Q–Q plots of the Pearson residuals

In this section, we illustrate RQRs in comparison to Pearson residuals for diagnosing six GLMMs for a single dataset generated with ZMNB models with scenario 4 parameter settings (low count, low ZP). RQRs are calculated by our created function rqr.glmmtmb or rqr.hurdle.glmmtmb. Figure 1 depicts the results simulated from ZMNB model when $n=400$. The panels in the first column display the RQRs versus fitted values, from which we can see that residuals from model ZMNB and ZINB are randomly scattered around $y=0$ without any discernible pattern. The standardized residuals for those two models are within -3 to 3, which indicates the ZINB model has similar fitting results as the ZMNB model, and both fit the data well. However, the RQRs for the models ZMP and ZIP are not evenly distributed around $y=0$, suggesting that ZMP and ZIP models do not fit the data well. This indicates that ZMP and ZIP models fail to model the over-dispersion adequately. The RQRs from the NB model show a decreased pattern, and the RQRs from the Poisson model are clustered at the top and bottom, indicating that the Poisson model could not handle over-dispersion and excessive zeros well. The panels in the second column display the scatter plots of Pearson residuals versus fitted values, which indicates that the Pearson residuals fail to provide meaningful information regarding the GOF of the models, which is not surprising, as Pearson residuals are theoretically not normally distributed for count regression.

The normality of the RQRs is examined using Q–Q normality plots, as shown in the panels of the third column of Fig. 1. The points in the Q–Q plots for ZMNB and ZINB align closely to the straight line with a slope of 1, which indicates these two models fit the data reasonably well. In contrast, the Q–Q plots for the ZMP, ZIP, and Poisson models are depicted as two separate lines with a substantial gap, which clearly indicates that the distributional assumptions for these models are not consistent with the true data. The points of the QQ plot of RQRs for the NB model shown in Fig. 1 follow more closely along the straight line than the ZMP, ZIP, and Poisson models, since NB distributions have heavy tails at two sides when $\theta$ is small. However, the misfit of NB models for the zero-inflated data can still be clearly identified in the scatter plot of RQRs. This demonstrates the advantage of examining the scatter plots of RQRs against fitted values for diagnosing model fit. Q–Q plots for the Pearson residuals are depicted in the panels of the fourth column of Fig. 1, all of which are curves, which poses a challenge for visually checking the model fit. The performance of RQRs is also examined when data are simulated from ZMP, ZINB and ZIP models, respectively, as shown in Additional file 1: Figs. S1, S2 and S3. The results indicate that RQRs are able to diagnose the model fit while Pearson residuals provide very limited information for checking the model fit.

#### Results of GOF tests based on RQRs with multiple simulated datasets

This section presents the results of examining the performance of RQRs for diagnosing the GOF of the regression models based on 3000 replicated datasets from the true model. The Shapiro–Wilk (SW) normality test for the residuals is used as the overall GOF test. When the model is true, the *p* values obtained from SW normality test are expected to be a uniform distribution. Additional file 1: Fig. S4 shows that when the true model is the ZMNB model, the *p* values obtained from the SW normality test of RQRs for ZMNB and ZINB are uniformly distributed in all four scenarios. Similar to the results presented in Section “Illustration of model diagnostics with RQRs for a single dataset”, both ZMNB and ZINB models perform well when data are simulated from ZMNB, while the rest four models fail to adequately capture the over-dispersion or zero-inflation. Additional file 1: Fig. S5 indicates that the *p* values of the SW normality test for the Pearson residuals are all concentrated around zero; therefore, Pearson residuals fail to distinguish the true and wrong models.

We further investigate the type I error rate of the SW normality test (probability of rejecting the true model) for RQRs and Pearson residuals at varying sample sizes, $n=50,100,200$, and 400. Ideally, the type I error of the SW normality test should be around 0.05. Table 1 presents the probability of rejecting the model when the 3000 replicated datasets of size $n=100$ are simulated from ZMNB, ZINB, ZMP, and ZIP, respectively. In each scenario, we summarize the zero proportion, 5%, 50%, and 95% quantiles of non-zero counts over the 3000 replicated datasets. We also summarize the number of converged model fittings for the 3000 replicated datasets, shown as *N* in the last columns of Tables 1 and 2. When the true model is ZMNB or ZINB, the type I error rates are close to 0.05, and the probabilities of rejecting the ZMP, ZIP, NB and Poisson models are all very high, indicating that RQRs are able to identify the misspecified models. When the true model is ZMP or ZIP, the type I error rates for ZMNB, ZINB, ZMP, and ZIP models are around 0.05 under different scenarios. In each scenario, the probabilities of rejecting the NB model are above 0.17, and the probabilities of rejecting the Poisson model are 1, suggesting RQRs can detect that NB and Poisson models are not appropriate for modelling zero-inflated data. In particular, the Poisson model is unable to model excess zeros. Moreover, zero proportion and the dispersion of the positive count data do not influence the type I error rates for the correctly specified model. Note that the convergence rates for all the models are higher when the true model is ZMNB or ZINB, as compared to the scenarios when data are generated from ZMP or ZIP model. Such difference is attributed to the need to use an extremely large $\theta$ of NB distribution for approximating the Poisson distribution; this often breaks down the model fitting with the package glmmTMB. However, this problem may be solved by other GLMM packages.

When the sample size $n=50, 200$ and 400 (Additional file 1: Tables S1, S2; Table 2), the results are consistent with result when sample size $n=100$. Hence, the type I error rate is not affected by the sample size. However, the convergence rate decreases when the sample size decreases. Only about 100 model fittings converged when $n=100$ compared to about 900 converged replicated datasets when $n=400$ in the scenario when data are simulated from the ZMP model. The convergence issue is more likely to occur when the sample size is too small to estimate the parameters reliably.

In comparison to the RQRs, Pearson residuals cannot differentiate the true and wrong models, as displayed in Additional file 1: Tables S3–S6. The SW normality tests based on Pearson residuals for all the models have high probabilities of rejecting models regardless of the sample sizes, zero proportion, and scale of count data. For example, the probabilities of rejecting the true model ZMNB are all equals to 1 under all scenarios and different sample sizes. Therefore, Pearson residuals are useless compared with RQRs for testing the overall GOF of the methods.

**Table 1:** Probability of rejecting the normality of RQRs based on SW normality test when $n = 100$

Scenario	ZP	$Q_{0.05}$	$Q_{0.5}$	$Q_{0.95}$	ZMNB*	ZINB$\dagger$	ZMP	ZIP	NB	Poisson	N
1	59	320	1075	2711	0.04	0.05	1	1	0.29	1	1604
2	59	32	109	275	0.04	0.03	1	0.99	0.18	1	1312
3	31	302	1078	2800	0.04	0.04	1	1	0.84	1	1720
4	31	29	107	280	0.05	0.05	1	0.99	0.75	1	1552

Scenario	ZP	$Q_{0.05}$	$Q_{0.5}$	$Q_{0.95}$	ZMNB$\dagger$	ZINB*	ZMP	ZIP	NB	Poisson	N
1	58	317	1079	2727	0.04	0.04	1	1	0.28	1	1580
2	58	31	108	274	0.04	0.04	1	1	0.17	1	1276
3	31	301	1071	2783	0.05	0.04	1	1	0.85	1	1691
4	31	30	107	279	0.04	0.04	1	1	0.73	1	1535

Scenario	ZP	$Q_{0.05}$	$Q_{0.5}$	$Q_{0.95}$	ZMNB$\dagger$	ZINB$\dagger$	ZMP*	ZIP$\dagger$	NB	Poisson	N
1	55	807	1036	1335	0.03	0.04	0.05	0.04	0.42	1	398
2	56	76	103	138	0.04	0.06	0.06	0.04	0.25	1	339
3	28	768	1008	1320	0.04	0.05	0.05	0.05	0.93	1	633
4	30	73	101	138	0.03	0.04	0.06	0.03	0.83	1	405

Scenario	ZP	$Q_{0.05}$	$Q_{0.5}$	$Q_{0.95}$	ZMNB$\dagger$	ZINB$\dagger$	ZMP$\dagger$	ZIP*	NB	Poisson	N
1	55	793	1020	1318	0.03	0.05	0.03	0.05	0.42	1	390
2	54	74	102	138	0.04	0.04	0.06	0.05	0.30	1	338
3	28	781	1020	1341	0.04	0.03	0.04	0.04	0.92	1	617
4	29	72	102	139	0.03	0.04	0.04	0.04	0.84	1	451

*Represents the true data generating model and ${\dagger}$ represents the models that theoretically contain or are very close to the true data generating model. ZP is the average zero percentages. The three columns labelled by $Q_\alpha$ show the average of the quantiles of non-zero counts for three $\alpha$. N is the number of converged model fittings over 3000 replicated datasets

**Table 2:** Probability of rejecting the normality of RQRs based on SW normality test when $n = 400$

Scenario	ZP	$Q_{0.05}$	$Q_{0.5}$	$Q_{0.95}$	ZMNB*	ZINB$\dagger$	ZMP	ZIP	NB	Poisson	N
1	60	285	1068	2808	0.03	0.03	1	1	0.56	1	2475
2	59	28	108	287	0.04	0.04	1	1	0.50	1	2199
3	30	281	1072	2850	0.04	0.05	1	1	0.95	1	2596
4	29	27	107	287	0.04	0.04	1	1	0.90	1	2472

Scenario	ZP	$Q_{0.05}$	$Q_{0.5}$	$Q_{0.95}$	ZMNB$\dagger$	ZINB*	ZMP	ZIP	NB	Poisson	N
1	60	285	1070	2825	0.04	0.04	1	1	0.57	1	2451
2	60	28	108	286	0.03	0.03	1	1	0.49	1	2212
3	30	280	1069	2843	0.04	0.04	1	1	0.95	1	2613
4	29	27	107	287	0.04	0.04	1	1	0.90	1	2485

Scenario	ZP	$Q_{0.05}$	$Q_{0.5}$	$Q_{0.95}$	ZMNB$\dagger$	ZINB$\dagger$	ZMP*	ZIP$\dagger$	NB	Poisson	N
1	59	777	1012	1315	0.04	0.04	0.05	0.04	0.64	1	906
2	59	74	102	138	0.04	0.03	0.04	0.04	0.57	1	839
3	29	769	1011	1334	0.05	0.05	0.06	0.06	0.97	1	1065
4	29	73	102	139	0.04	0.05	0.06	0.04	0.94	1	960

Scenario	ZP	$Q_{0.05}$	$Q_{0.5}$	$Q_{0.95}$	ZMNB$\dagger$	ZINB$\dagger$	ZMP$\dagger$	ZIP*	NB	Poisson	N
1	59	782	1015	1318	0.04	0.04	0.04	0.05	0.63	1	954
2	59	74	103	139	0.04	0.04	0.04	0.05	0.58	1	816
3	29	769	1015	1340	0.04	0.04	0.06	0.05	0.97	1	1015
4	28	73	102	139	0.04	0.04	0.05	0.05	0.95	1	936

*Represents the true data generating model and $\dagger$ represents the models that theoretically contain or are very close to the true data generating model. The three columns labelled by $Q_\alpha$ show the average of the quantiles of non-zero counts for three $\alpha$. ZP is the average zero percentage. N is the number of converged fittings over 3000 replicated datasets

## Application to a real human microbiome dataset

In this section, a real human microbiome dataset will be introduced. We apply various models discussed previously to this dataset and use RQRs to test the GOF of all models.

### Data sources and descriptions

**Fig. 2:**
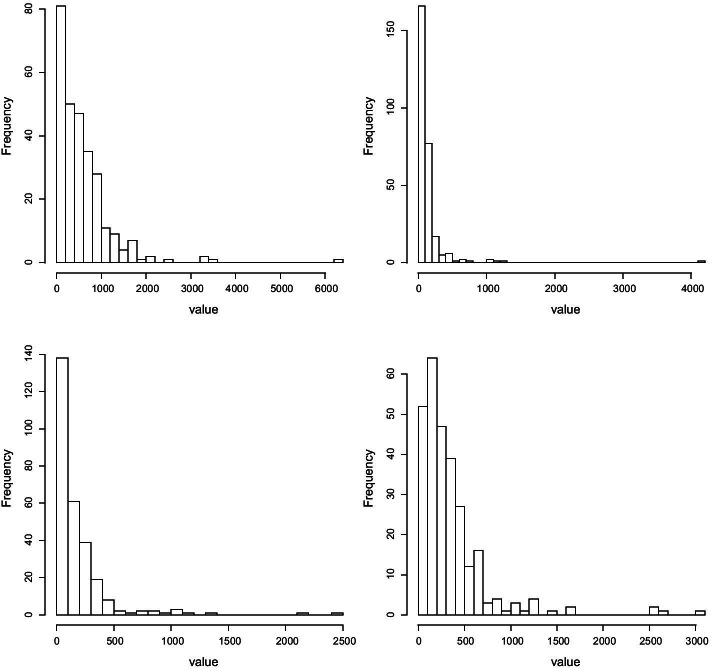
Histograms of the OTU counts of four selected genera

As a response to the epidemic of worldwide obesity, efforts to identify the relationship between host and environmental factors and energy balance have increased. Comparisons of the distal gut microbiota of genetically obese mice and their lean littermates have revealed that obesity is associated with two dominant bacterial divisions, i.e., the Bacteroidetes and the Firmicutes [48]. The human distal gut harbours a vast ensemble of microbes helping to break down otherwise indigestible material. It is often of interest to investigate the relationship between gut microbial ecology and body fat in humans [49]. Each distinct microbe species can be assigned to a diverse taxonomic rank based on shared characteristics, including species, genus, family, order, class, phylum, kingdom, and domain. The OTU data used in our application were generated at the genus level, which is the commonly used OTU level for microbiome sequencing analysis, and there are 14 different genera in total [50]. Each sample consists of 154 individuals, and we characterize individuals into 31 monozygotic (MZ) twin pairs, 23 dizygotic (DZ) twin pairs and 46 mothers. Twins were between 21 and 32 years old and were of European (EA) or African (AA) ancestry, respectively. Individuals were classified as obese/overweight if body mass index (BMI) $\ge$ 25, or lean if BMI < 25. Fecal samples were frozen immediately after they were produced for extracting the DNAs of the bacteria, then the 16S rRNA sequencing method was used to group the bacteria into different OTUs with a sequence identity threshold of 97% [11]. Two subjects were dropped from samples for quality control. Among the rest of the 152 individuals, 34 were measured once, and 118 were measured twice (time point 1 and time point 2) for fecal samples. There are 281 OTU measures on the genus level in total. For each measurement, OTU count at each genus level, as well as the total number of reads per measure, were recorded. Figure 2 shows the histograms of the four genera selected from the data for the purpose of illustration of the distribution of the OTU measures, which all exhibit right skewness.

### Model checking with the RQR method

In this analysis, ancestry and obesity were selected as the host factors while age and family as the random factors. Then, ZMNB, ZMP, ZINB, ZIP, NB and Poisson models were fitted to each of the 14 genus-level OTUs. First, we fitted Poisson and NB models to the original dataset. However, the model checking results based on examining the normality of their RQRs showed that these models do not fit the original data very well (results shown in [51]). The OTU counts at the genus level contain very few actual zeros. Therefore, zero-inflated models cannot fit the original data better. Considering that small OTU counts at the genus level are likely caused by the mismatching in sequence alignment of reads, we truncated the OTU counts to be zero when their values are less than 10 for most genera except the following four genera. The truncation thresholds for Bacteroides, Ruminococcus,Faecalibacterium and Lachnospiraceae are set to be 50, 50, 100, and 150, respectively. We will present the model diagnostics results for the truncated datasets.

**Fig. 3:**
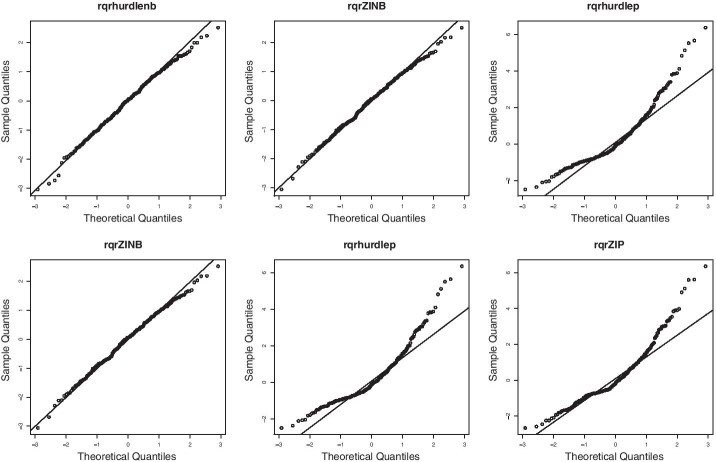
Q–Q plots of RQRs of six models fitted to Euba OTU data from the Twin Study. The names of models are as follows: ZMNB (top left), ZINB (top middle), ZMP (top right), ZIP (bottom left), NB (bottom middle), Poisson (bottom right)

Figure 3 shows the Q–Q plots of the RQRs of six models fitted to Euba OTU counts. We see that the Q–Q plots of the RQRs of the ZMNB model and the ZINB model fall along a straight line with a slope of 1 and just a few points slightly deviating from the diagonal line, which indicates that RQRs are normally distributed. The Q–Q plots for the other four models exhibit curvature patterns. Therefore, these Q–Q plots show that only ZMNB and ZINB appear to fit the dataset well.

Table 3 shows the *p* values for the SW normality test of RQRs for all 14 OTUs at the genus level. For easy visual inspection of RQRs, we sort the genera by the test *p* values of the ZINB model. The first column lists 14 different genera in the twin study OTU data. If the *p* value for the SW normality test is less than 0.05, the model may not fit the data well. RQRs contain randomness. As a result, we calculate the mean of the SW test *p* values based on RQRs by replicating RQRs 100 times. As shown in Table 3, the ZMNB and ZINB models provide reasonable fits to this data with all SW *p* values greater than 0.05. However, the SW *p* values for the ZMP, ZIP, NB, Poisson, NB, and Poisson models are mostly very small (except the *p* value of NB for Blau). These small SW test *p* values indicate that these models do not fit the data well. We also use the Akaike information criterion (AIC) to compare the six models. Table 4 presents the AIC values of all of the six models. The ZMNB and ZINB models also have smaller AIC values compared to other models. The model comparison results based on AIC are consistent with the GOF test results based on RQRs.

**Table 3:** *P* values for the SW normality test of RQRs for the Twin Study

Genus	ZMNB	ZINB	ZMP	ZIP	NB	Poisson
Bact	0.052	0.034	$<10^{-19}$	$<10^{-19}$	$<10^{-16}$	$<10^{-18}$
Lach..g	0.072	0.074	$<10^{-16}$	$<10^{-15}$	$<10^{-3}$	$<10^{-11}$
Faec	0.083	0.107	$<10^{-17}$	$<10^{-18}$	$<10^{-17}$	$<10^{-15}$
Rumi	0.232	0.285	$<10^{-19}$	$<10^{-19}$	$<10^{-6}$	$<10^{-12}$
Rumi.1	0.238	0.366	$<10^{-16}$	$<10^{-16}$	$<10^{-10}$	$<10^{-11}$
Blau	0.251	0.104	$<10^{-10}$	$<10^{-10}$	0.087	$<10^{-12}$
Erys	0.344	0.258	$<10^{-16}$	$<10^{-17}$	$<10^{-4}$	$<10^{-7}$
Alis	0.344	0.352	$<10^{-16}$	$<10^{-16}$	$<10^{-9}$	$<10^{-7}$
Euba	0.461	0.539	$<10^{-15}$	$<10^{-15}$	$<10^{-10}$	$<10^{-6}$
Lach	0.521	0.358	$<10^{-9}$	$<10^{-10}$	$<10^{-10}$	$<10^{-5}$
Oscil	0.535	0.606	$<10^{-15}$	$<10^{-15}$	$<10^{-9}$	$<10^{-5}$
Prev	0.605	0.269	$<10^{-17}$	$<10^{-17}$	$<10^{-4}$	$<10^{-12}$
Rose	0.627	0.613	$<10^{-13}$	$<10^{-14}$	$<10^{-6}$	$<10^{-13}$
Copr	0.752	0.721	$<10^{-13}$	$<10^{-14}$	$<10^{-8}$	$<10^{-6}$

The rows were sorted according to the *p* values of ZMNB models in an ascending order

**Table 4:** AIC of the competing models for modeling the OTU data in the Twin Study

Genus	ZMNB	ZINB	ZMP	ZIP	NB	Poisson
Bact	3698.20	3689.41	29,583.11	30,276.44	3954.58	52,572.41
Lach..g	1096.77	1156.08	Inf	5715.61	1317.49	18,248.69
Faec	3328.72	3263.50	13,524.32	14,132.08	3620.03	29,430.38
Rumi	1597.93	1700.08	4495.07	5007.55	1896.48	13,263.94
Rumi.1	2432.82	2501.87	6993.35	7536.15	2703.78	13,199.43
Blau	3425.68	3401.05	18,403.01	18,946.44	3396.90	19,206.77
Erys	1530.03	1642.23	4129.93	4585.44	1782.82	9082.96
Alis	2159.41	2267.34	4768.40	5300.08	2418.27	9055.12
Euba	2108.20	2123.68	3617.31	4034.78	2292.49	6937.54
Lach	2089.01	2069.09	2325.09	2702.97	2262.49	5286.85
Oscil	1941.61	2044.71	3629.43	4104.53	2218.59	7624.30
Prev	1261.25	1364.03	3757.93	4221.34	1472.21	41,117.31
Rose	3234.63	3272.57	18,000.67	18,547.76	3340.65	21,270.47
Copr	2848.91	2829.24	6486.43	6949.32	2914.87	8750.86

## Discussion and conclusion

Model checking is critical to control the FDR at a nominal level in differential abundance analysis for sequencing count data. In this paper, we conduct large-scale simulation studies to investigate the performance of the RQRs diagnosing zero-inflated GLMMs, which are often applied to model sequencing count data. Our simulation studies show that the type I error rates of the GOF tests with RQRs are very close to the nominal level. In addition, the scatter plots and Q–Q plots of RQRs are useful in discerning the true and wrong models. We also apply the RQRs to diagnose six GLMMs in a real microbiome data analysis. The results show that the OTU counts at the genus level of this dataset after a truncation treatment can be modelled well by zero-inflated and zero-modified NB models. In conclusion, RQR is an excellent tool for diagnosing GLMMs for zero-inflated count data, such as the sequencing count data arising in microbiome studies. In the Additional file 1, two generic R functions, called rqr.glmmtmb and rqr.hurdle.glmmtmb, are provided for calculating the RQRs given fitting outputs of the R package glmmTMB.

The application of the RQR method in a real microbiome dataset shows that ZINB and ZMNB can provide adequate fits to the OTU counts after truncation of small values. This conclusion may not be generalized to all microbiome datasets. However, it is of interest to conduct the model diagnostics with RQRs to the ZINB and ZMNB models fitted to a large number of sequencing count datasets. In addition to the zero-inflated GLMMs for count data, the RQR method can also be applied to other two-part models, such as zero-inflated beta or zero-inflated log-normal models [52, 53], for which the randomization needs only to be applied to the observed zeros. This is an interesting research topic to pursue in the future.

## Supplementary Information

**Additional file 1**. Additional Simulation Results and RQR Functions for R package ‘glmmTMB’.

## Data Availability

Genotyping data and summary statistics are available from http://www.gemproject.ca/.

## Abbreviations

CDF: Cumulative distribution function
GOF: Goodness-of-fit
GLM: Generalized linear model
GLMM: Generalized linear mixed model
OTU: Operational Taxonomic Units
PMF: Probability mass function
RQR: Randomized quantile residuals
SW: Shaprio–Wilk
Q–Q plots: Quantile–quantile plots
NB: Negative binomial
ZMP: Zero-modified Poisson/Hurdle Poisson
ZMNB: Zero-modified negative binomial/ Hurdle NB
ZIP: Zero-inflated Poisson
ZINB: Zero-inflated negative binomial
