# Supplementary material for: Randomized quantile residuals for diagnosing zero-inflated generalized linear mixed models with applications to microbiome count data
Source: BMC Bioinformatics. 2021 Nov 25;22:564. doi: 10.1186/s12859-021-04371-6 (PMC8620156; doi:10.1186/s12859-021-04371-6)
Supplement: Supplementary file 1 — Additional file 1. Additional Simulation Results and RQR Functions for R package ‘glmmTMB’. [file 12859_2021_4371_MOESM1_ESM.pdf]

Supplementary Materials for Randomized Quantile Residuals for Diagnosing Zero-Inflated Generalized Linear Mixed Models with Applications to Microbiome Count Data

1 Supplementary Figures

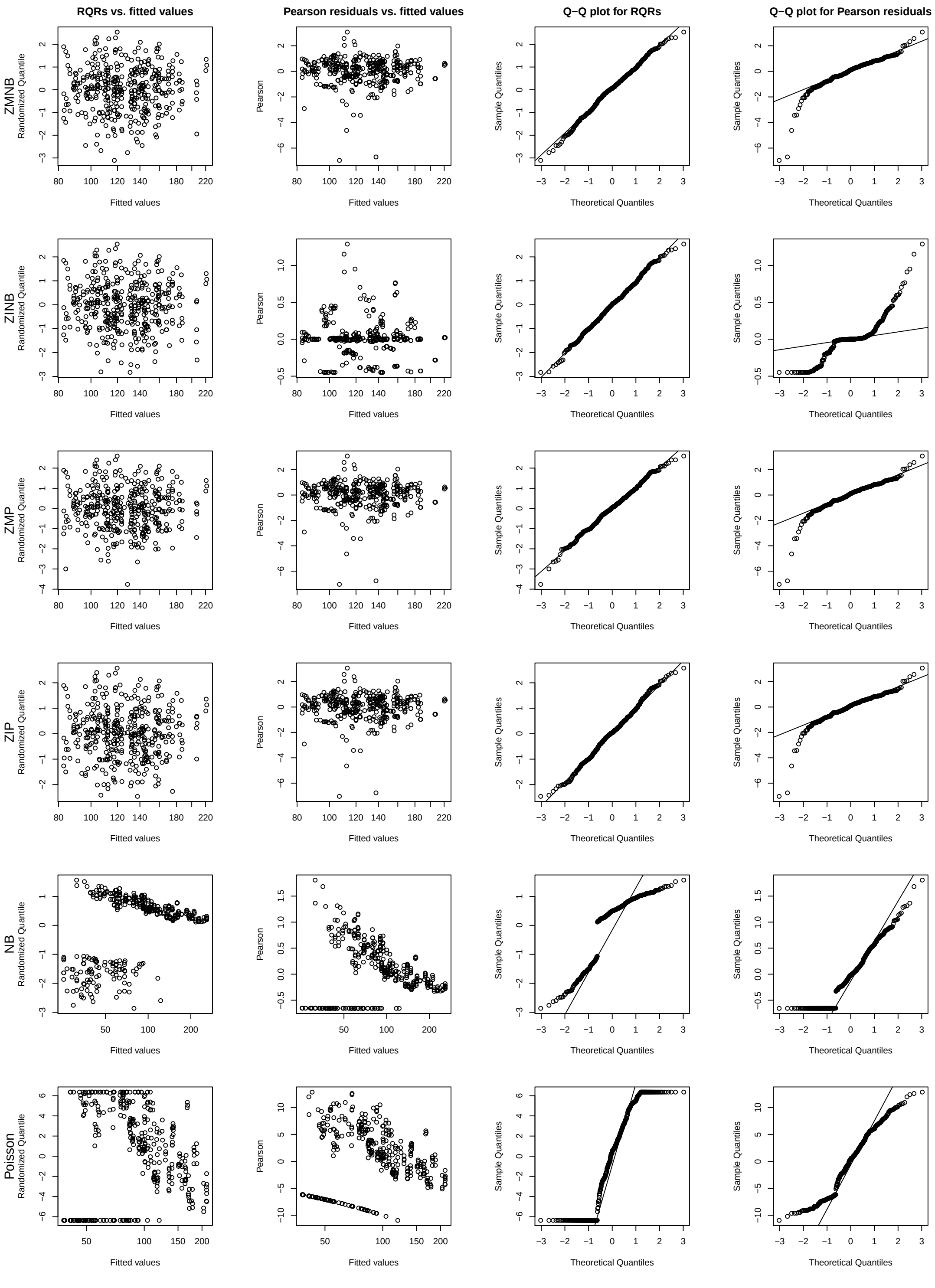

Figure S1: Model diagnostics for a single dataset of $n = 400$ samples simulated from a ZMP model with scenario 4 parameter settings (low count, low zero proportion). The panels in the first column are the scatter plots of the RQRs vs. fitted values. The panels in the second column depict the scatter plots of the Pearson residuals vs. fitted values. The panels in the third column present the Q-Q plots for RQRs. The panels in the fourth column present the Q-Q plots of the Pearson residuals.

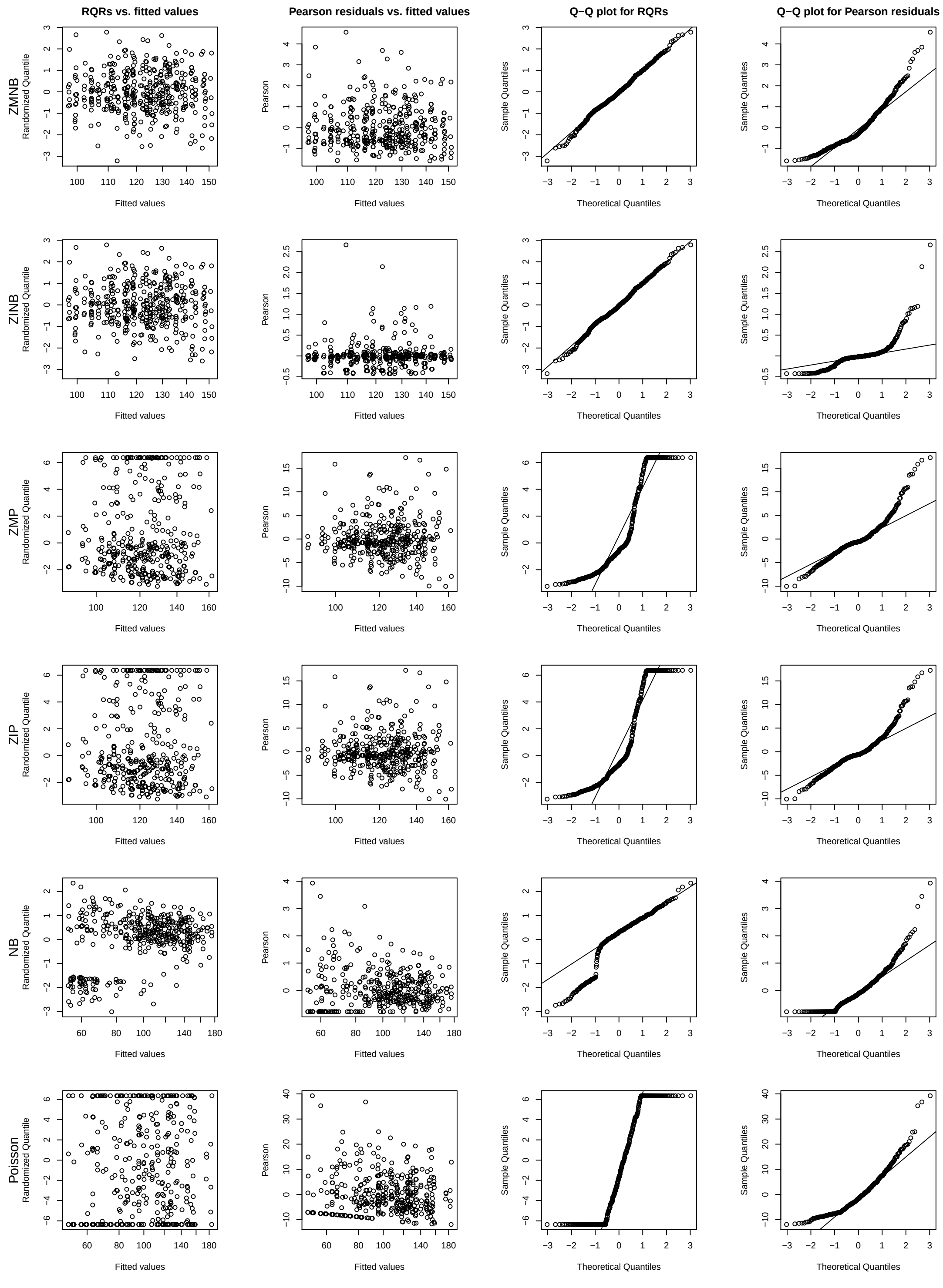

Figure S2: Model diagnostics for a single dataset of $n = 400$ samples simulated from a ZINB model with scenario 4 parameter settings (low count, low zero proportion). The panels in the first column are the scatter plots of the RQRs vs. fitted values. The panels in the second column depict the scatter plots of the Pearson residuals vs. fitted values. The panels in the third column present the Q-Q plots for RQRs. The panels in the fourth column present the Q-Q plots of the Pearson residuals.

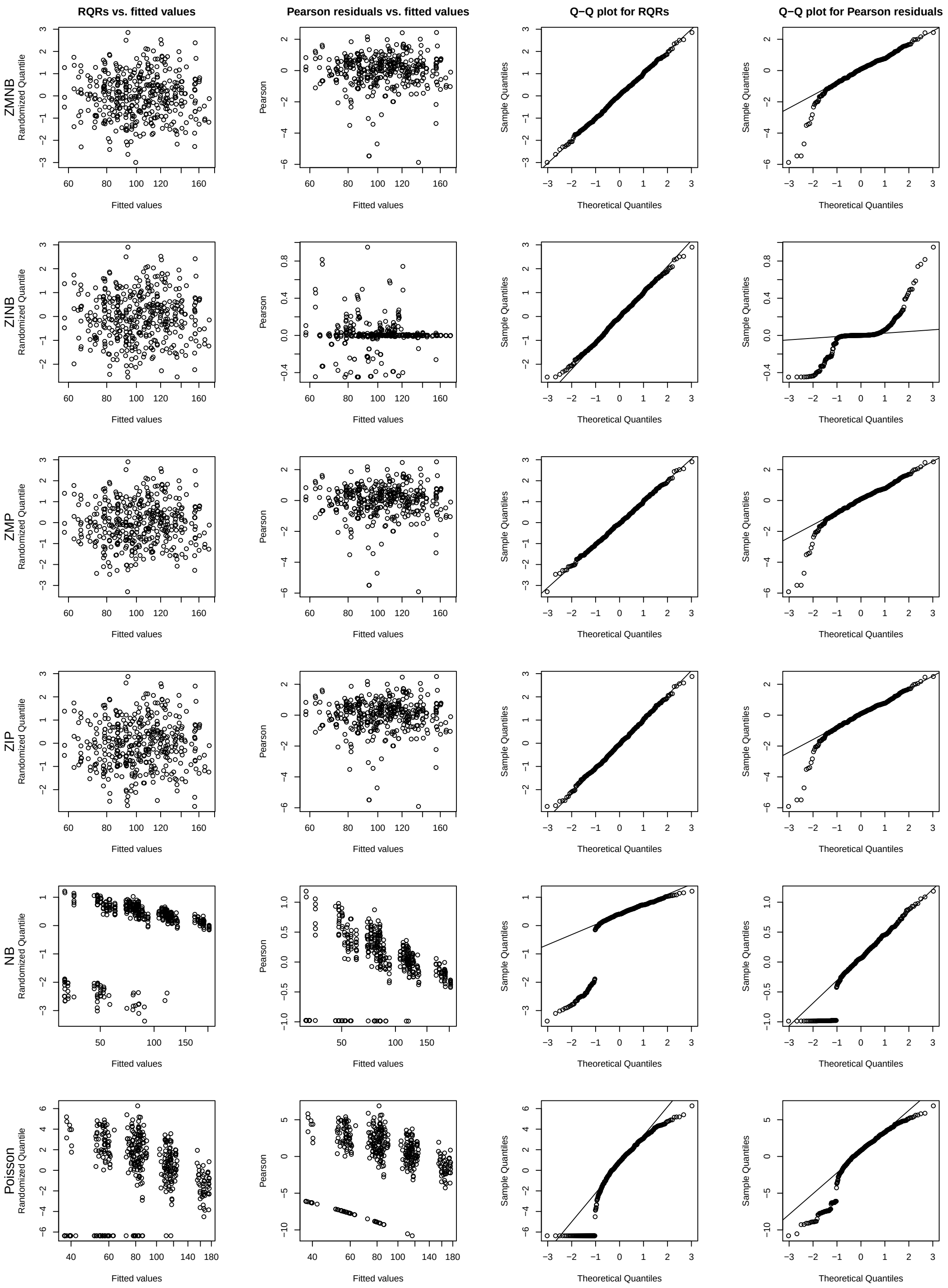

Figure S3: Model diagnostics for a single dataset of $n = 400$ samples simulated from a ZIP model with scenario 4 parameter settings (low count, low zero proportion). The panels in the first column are the scatter plots of the RQRs vs. fitted values. The panels in the second column depict the scatter plots of the Pearson residuals vs. fitted values. The panels in the third column present the Q-Q plots for RQRs. The panels in the fourth column present the Q-Q plots of the Pearson residuals.

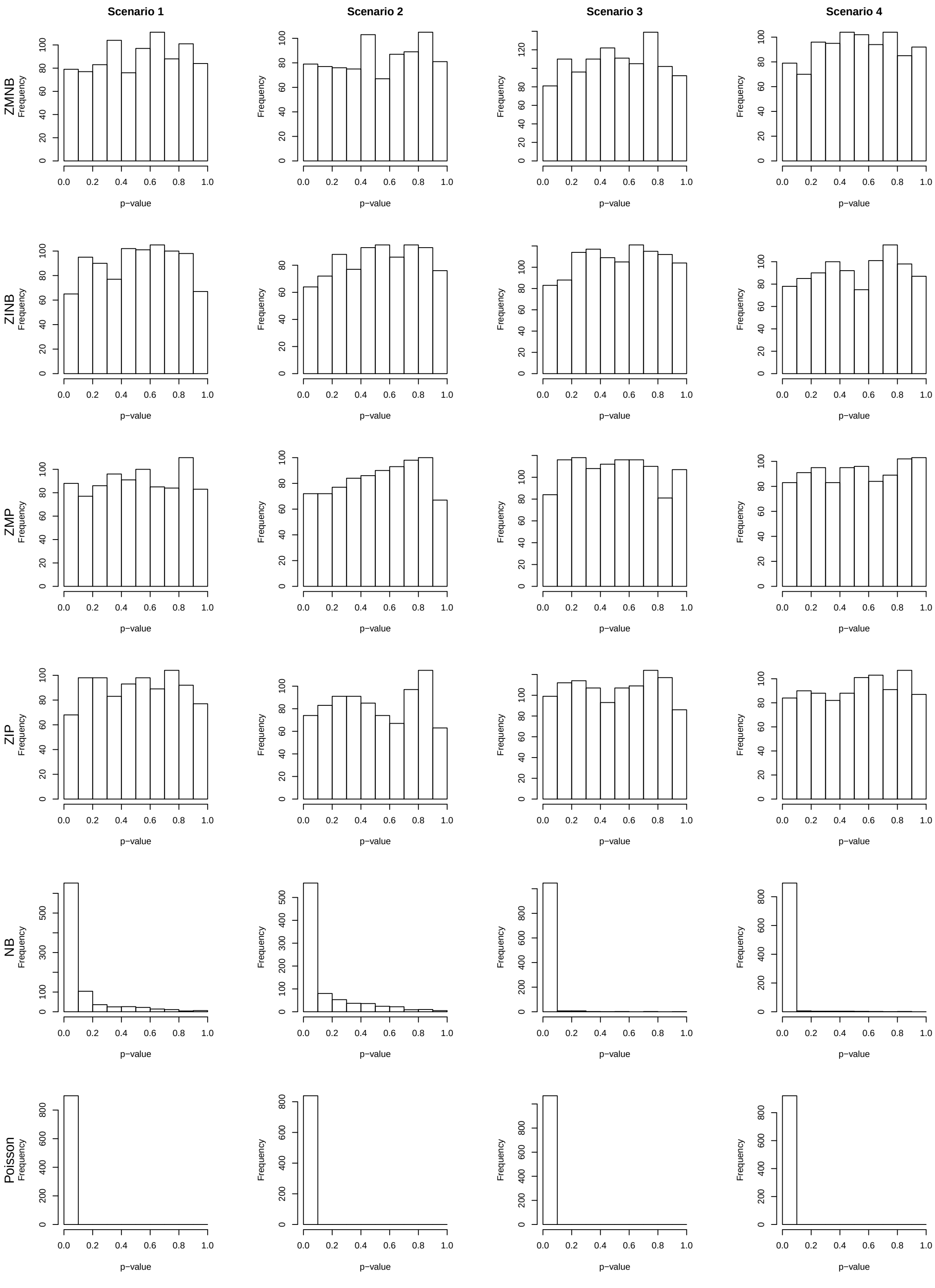

Figure S4: Histograms of the p-values for the SW normality test of RQRs for ZMNB (1st row), ZINB (2nd row), ZMP (3rd row), ZIP (4th row), NB (5th row) and Poisson (6th row) models when the dataset is simulated from ZMP model (n=400) under different scenarios. The panels in the first column depict scenario 1. The panels in the second column depict scenario 2. The panels in the third column represent scenario 3. The panels in the fourth column represent scenario 4.

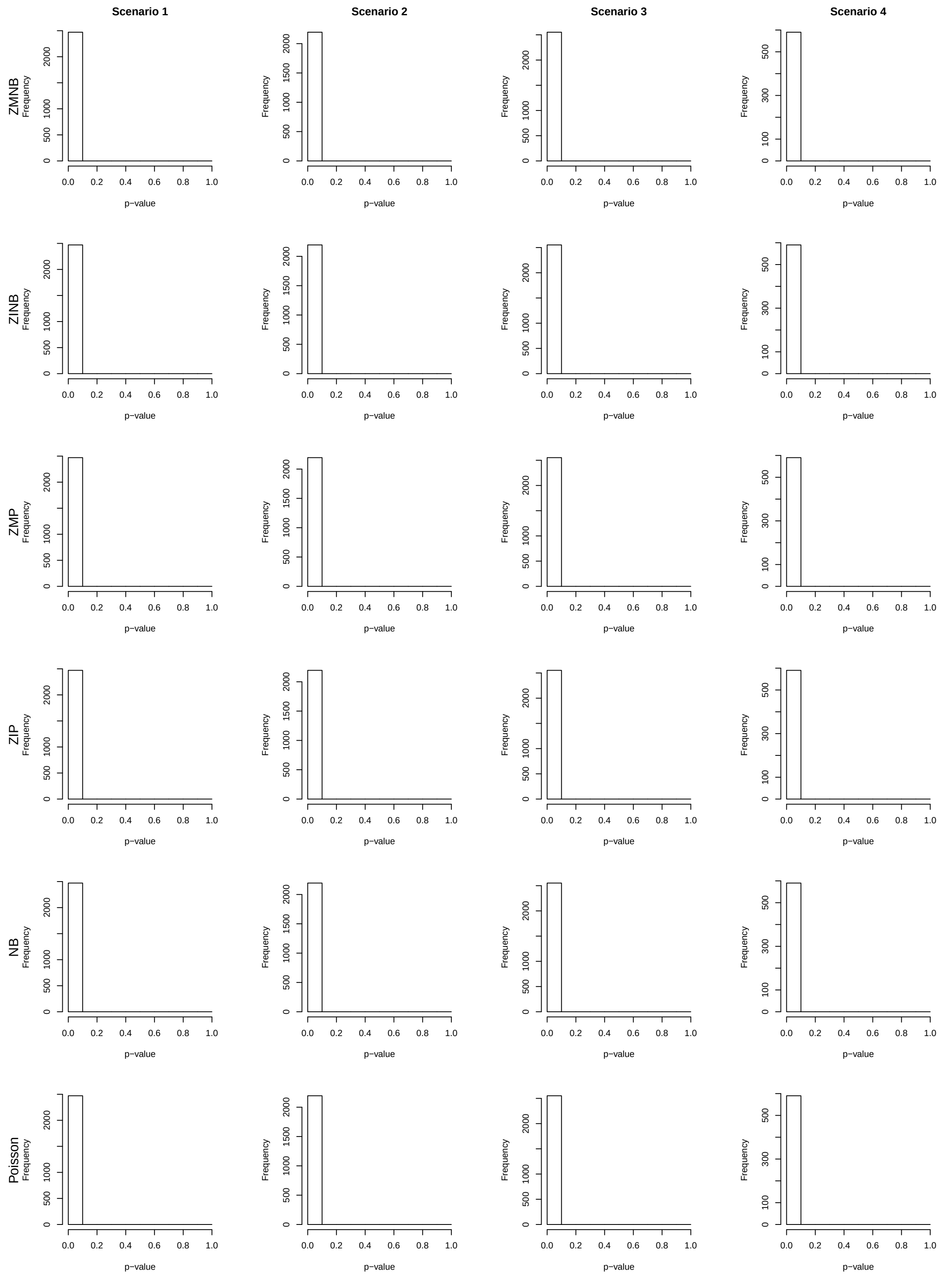

Figure S5: Histograms of the p-values for the SW normality test of Pearson residuals for ZMNB (1st row), ZINB (2nd row), ZMP (3rd row), ZIP (4th row), NB (5th row) and Poisson (6th row) models when the dataset is simulated from ZMNB model (n=400) under different scenarios. The panels in the first column depict scenario 1. The panels in the second column depict scenario 2. The panels in the third column represent scenario 3. The panels in the fourth column represents scenario 4.

2 Supplementary Tables

Table S1: Probability of rejecting the normality of RQRs based on SW normality test when $n = 50$. * represents the true data generating model and † represents the models theoretically equivalent or very close to the true data generating model. ZP is the average zero percentages. The three columns labelled by Q_α show the average of the quantiles of non-zero counts for three α. N is the number of converged model fittings over 3000 replicated datasets.

Scenario	ZP	$Q_{0.05}$	$Q_{0.5}$	$Q_{0.95}$	ZMNB*	ZINB†	ZMP	ZIP	NB	Poisson	N
1	56	357	1096	2641	0.04	0.05	0.99	0.99	0.30	1.00	768
2	56	35	111	268	0.05	0.05	0.88	0.87	0.18	1.00	600
3	33	326	1081	2742	0.05	0.04	1.00	1.00	0.76	1.00	875
4	32	32	109	281	0.05	0.05	0.89	0.88	0.61	1.00	704
Scenario	ZP	$Q_{0.05}$	$Q_{0.5}$	$Q_{0.95}$	ZMNB†	ZINB*	ZMP	ZIP	NB	Poisson	N
1	55	355	1077	2611	0.04	0.04	0.99	0.99	0.28	1.00	783
2	56	35	108	262	0.05	0.06	0.86	0.86	0.14	1.00	589
3	34	326	1080	2732	0.04	0.05	1.00	1.00	0.78	1.00	912
4	34	32	108	272	0.05	0.06	0.85	0.86	0.57	1.00	720
Scenario	ZP	$Q_{0.05}$	$Q_{0.5}$	$Q_{0.95}$	ZMNB†	ZINB†	ZMP*	ZIP†	NB	Poisson	N
1	50	770	989	1261	0.06	0.03	0.06	0.03	0.44	1.00	97
2	49	74	100	134	0.01	0.02	0.01	0.02	0.36	1.00	91
3	31	785	1016	1320	0.04	0.03	0.04	0.04	0.86	1.00	240
4	30	74	102	138	0.02	0.02	0.02	0.02	0.79	1.00	125
Scenario	ZP	$Q_{0.05}$	$Q_{0.5}$	$Q_{0.95}$	ZMNB†	ZINB†	ZMP†	ZIP*	NB	Poisson	N
1	50	790	1004	1305	0.04	0.01	0.02	0.013	0.45	1.00	119
2	49	75	102	136	0.04	0.10	0.03	0.08	0.35	1.00	78
3	31	774	1002	1293	0.05	0.05	0.03	0.04	0.88	1.00	211
4	31	71	99	137	0.05	0.02	0.04	0.05	0.74	1.00	157

Table S2: Probability of rejecting the normality of RQRs based on SW normality test when $n = 200$. * represents the true data generating model and † represents the models theoretically equivalent or very close to the true data generating model. ZP is the average zero percentages. The three columns labelled by Q_α show the average of the quantiles of non-zero counts for three α. N is the number of converged model fittings over 3000 replicated datasets.

Scenario	ZP	$Q_{0.05}$	$Q_{0.5}$	$Q_{0.95}$	ZMNB*	ZINB†	ZMP	ZIP	NB	Poisson	N
1	60	298	1077	2790	0.04	0.04	1.00	1.00	0.36	1.00	2081
2	59	29	108	282	0.04	0.04	1.00	1.00	0.25	1.00	1889
3	30	289	1074	2836	0.04	0.04	1.00	1.00	0.92	1.00	2253
4	28	27	107	285	0.04	0.04	1.00	1.00	0.83	1.00	2189
Scenario	ZP	$Q_{0.05}$	$Q_{0.5}$	$Q_{0.95}$	ZMNB†	ZINB*	ZMP	ZIP	NB	Poisson	N
1	59	317	1079	2727	0.04	0.04	1.00	1.00	0.28	1.00	1580
2	58	31	108	273	0.04	0.04	1.00	1.00	0.17	1.00	1276
3	31	391	1071	2783	0.05	0.04	1.00	1.00	0.85	1.00	1691
4	31	30	107	279	0.04	0.04	1.00	1.00	0.73	1.00	1535
Scenario	ZP	$Q_{0.05}$	$Q_{0.5}$	$Q_{0.95}$	ZMNB†	ZINB†	ZMP*	ZIP†	NB	Poisson	N
1	59	791	1025	1330	0.03	0.04	0.05	0.04	0.44	1.00	705
2	57	74	101	137	0.04	0.04	0.03	0.04	0.33	1.00	576
3	29	776	1019	1342	0.04	0.03	0.04	0.04	0.96	1.00	834
4	29	72	101	139	0.06	0.04	0.04	0.05	0.88	1.00	804
Scenario	ZP	$Q_{0.05}$	$Q_{0.5}$	$Q_{0.95}$	ZMNB†	ZINB†	ZMP†	ZIP*	NB	Poisson	N
1	58	789	1018	1315	0.05	0.04	0.03	0.05	0.45	1.00	718
2	57	74	103	139	0.04	0.05	0.04	0.05	0.38	1.00	521
3	29	766	1005	1326	0.05	0.05	0.04	0.03	0.94	1.00	865
4	29	73	102	139	0.04	0.04	0.06	0.04	0.90	1.00	801

Table S3: Probability of rejecting the normality of Pearson residuals based on SW normality test when $n = 50$. * represents the true data generating model. ZP is the average zero percentages. The three columns labelled by Q_α show the average of the quantiles of non-zero counts for three α. N is the number of converged model fittings over 3000 replicated datasets.

Scenario	ZP	$Q_{0.05}$	$Q_{0.5}$	$Q_{0.95}$	ZMNB*	ZINB	ZMP	ZIP	NB	Poisson	N
1	56	357	1096	2641	0.95	1.00	1.00	1.00	1.00	1.00	768
2	56	35	111	268	0.94	1.00	0.97	0.97	1.00	1.00	600
3	33	326	1081	2742	0.85	0.99	0.97	0.97	1.00	0.96	875
4	32	32	109	281	0.85	1.00	0.89	0.88	1.00	0.95	704
Scenario	ZP	$Q_{0.05}$	$Q_{0.5}$	$Q_{0.95}$	ZMNB	ZINB*	ZMP	ZIP	NB	Poisson	N
1	55	355	1077	2611	0.96	1.00	0.99	0.99	1.00	0.99	783
2	56	35	108	262	0.95	1.00	0.98	0.98	1.00	0.99	589
3	34	326	1080	2732	0.84	1.00	0.97	0.97	1.00	0.96	912
4	34	32	108	272	0.83	1.00	0.88	0.88	1.00	0.96	720
Scenario	ZP	$Q_{0.05}$	$Q_{0.5}$	$Q_{0.95}$	ZMNB	ZINB	ZMP*	ZIP	NB	Poisson	N
1	50	770	989	1261	0.82	1.00	0.84	0.84	1.00	0.92	97
2	49	74	100	134	0.70	1.00	0.70	0.70	1.00	0.99	91
3	31	785	1016	1320	0.53	1.00	0.54	0.54	1.00	0.76	240
4	30	74	102	138	0.46	1.00	0.45	0.45	0.99	0.75	125
Scenario	ZP	$Q_{0.05}$	$Q_{0.5}$	$Q_{0.95}$	ZMNB	ZINB	ZMP	ZIP*	NB	Poisson	N
1	50	790	1004	1305	0.83	1.00	0.83	0.83	1.00	0.92	119
2	49	75	102	136	0.73	1.00	0.73	0.73	1.00	0.96	78
3	31	774	1002	1293	0.56	1.00	0.57	0.57	1.00	0.74	211
4	31	71	99	137	0.50	1.00	0.46	0.46	1.00	0.69	157

Table S4: Probability of rejecting the normality of Pearson residuals based on SW normality test when $n = 100$. * represents the true data generating model. ZP is the average zero percentages. The three columns labelled by Q_α show the average of the quantiles of non-zero counts for three α. N is the number of converged model fittings over 3000 replicated datasets.

Scenario	ZP	$Q_{0.05}$	$Q_{0.5}$	$Q_{0.95}$	ZMNB*	ZINB	ZMP	ZIP	NB	Poisson	N
1	59	320	1075	2711	1.00	1.00	1.00	1.00	1.00	1.00	1604
2	59	32	109	275	1.00	1.00	1.00	1.00	1.00	1.00	1312
3	31	302	1078	2800	1.00	1.00	1.00	1.00	1.00	1.00	1720
4	31	29	107	280	1.00	1.00	0.99	0.99	1.00	1.00	1552
Scenario	ZP	$Q_{0.05}$	$Q_{0.5}$	$Q_{0.95}$	ZMNB	ZINB*	ZMP	ZIP	NB	Poisson	N
1	58	317	1079	2727	1.00	1.00	1.00	1.00	1.00	1.00	1580
2	58	31	108	274	1.00	1.00	1.00	1.00	1.00	1.00	1276
3	31	301	1071	2783	1.00	1.00	1.00	1.00	1.00	1.00	1691
4	31	30	107	279	1.00	1.00	0.99	0.99	1.00	1.00	1535
Scenario	ZP	$Q_{0.05}$	$Q_{0.5}$	$Q_{0.95}$	ZMNB	ZINB	ZMP*	ZIP	NB	Poisson	N
1	55	807	1036	1335	0.98	1.00	0.98	0.98	1.00	1.00	398
2	56	76	103	138	0.96	1.00	0.96	0.96	1.00	1.00	339
3	28	768	1008	1320	0.86	1.00	0.86	0.86	1.00	0.99	633
4	30	73	101	138	0.03	0.74	0.72	0.73	1.00	0.97	405
Scenario	ZP	$Q_{0.05}$	$Q_{0.5}$	$Q_{0.95}$	ZMNB	ZINB	ZMP	ZIP*	NB	Poisson	N
1	55	793	1020	1318	0.98	1.00	0.98	0.98	1.00	1.00	390
2	54	74	102	138	0.95	1.00	0.95	0.95	1.00	1.00	338
3	28	781	1020	1341	0.83	1.00	0.83	0.83	1.00	0.97	617
4	29	72	102	139	0.69	1.00	0.69	0.69	1.00	0.98	451

Table S5: Probability of rejecting the normality of Pearson residuals based on SW normality test when $n = 200$. * represents the true data generating model. ZP is the average zero percentages. The three columns labelled by Q_α show the average of the quantiles of non-zero counts for three α. N is the number of converged model fittings over 3000 replicated datasets.

Scenario	ZP	$Q_{0.05}$	$Q_{0.5}$	$Q_{0.95}$	ZMNB*	ZINB	ZMP	ZIP	NB	Poisson	N
1	60	298	1077	2790	1.00	1.00	1.00	1.00	1.00	1.00	2081
2	59	29	108	282	1.00	1.00	1.00	1.00	1.00	1.00	1889
3	30	289	1074	2836	1.00	1.00	1.00	1.00	1.00	1.00	2253
4	28	27	107	285	1.00	1.00	1.00	1.00	1.00	1.00	2189
Scenario	ZP	$Q_{0.05}$	$Q_{0.5}$	$Q_{0.95}$	ZMNB	ZINB*	ZMP	ZIP	NB	Poisson	N
1	59	317	1079	2727	1.00	1.00	1.00	1.00	1.00	1.00	1580
2	58	31	108	273	1.00	1.00	1.00	1.00	1.00	1.00	1276
3	31	391	1071	2783	1.00	1.00	1.00	1.00	1.00	1.00	1691
4	31	30	107	279	1.00	1.00	1.00	1.00	1.00	1.00	1535
Scenario	ZP	$Q_{0.05}$	$Q_{0.5}$	$Q_{0.95}$	ZMNB	ZINB	ZMP*	ZIP	NB	Poisson	N
1	59	791	1025	1330	1.00	1.00	1.00	1.00	1.00	1.00	705
2	57	74	101	137	1.00	1.00	1.00	1.00	1.00	1.00	576
3	29	776	1019	1342	0.97	1.00	0.97	0.97	1.00	1.00	834
4	29	72	101	139	0.94	1.00	0.94	0.94	1.00	1.00	804
Scenario	ZP	$Q_{0.05}$	$Q_{0.5}$	$Q_{0.95}$	ZMNB	ZINB	ZMP	ZIP*	NB	Poisson	N
1	58	789	1018	1315	1.00	1.00	1.00	1.00	1.00	1.00	718
2	57	74	103	139	1.00	1.00	1.00	1.00	1.00	1.00	521
3	29	766	1005	1326	0.98	1.00	0.98	0.98	1.00	1.00	865
4	29	73	102	139	0.93	1.00	0.93	0.93	1.00	1.00	801

Table S6: Probability of rejecting the normality of Pearson residuals based on SW normality test when $n = 400$. * represents the true data generating model. ZP is the average zero percentages. The three columns labelled by Q_α show the average of the quantiles of non-zero counts for three α. N is the number of converged model fittings over 3000 replicated datasets.

Scenario	ZP	$Q_{0.05}$	$Q_{0.5}$	$Q_{0.95}$	ZMNB*	ZINB	ZMP	ZIP	NB	Poisson	N
1	60	285	1068	2808	1.00	1.00	1.00	1.00	1.00	1.00	2475
2	59	28	108	287	1.00	1.00	1.00	1.00	1.00	1.00	2199
3	30	281	1072	2850	1.00	1.00	1.00	1.00	1.00	1.00	2596
4	29	27	107	287	1.00	1.00	1.00	1.00	1.00	1.00	2472
Scenario	ZP	$Q_{0.05}$	$Q_{0.5}$	$Q_{0.95}$	ZMNB	ZINB*	ZMP	ZIP	NB	Poisson	N
1	60	285	1070	2825	1.00	1.00	1.00	1.00	1.00	1.00	2451
2	60	28	108	286	1.00	1.00	1.00	1.00	1.00	1.00	2212
3	30	280	1069	2843	1.00	1.00	1.00	1.00	1.00	1.00	2613
4	29	27	107	287	1.00	1.00	1.00	1.00	1.00	1.00	2485
Scenario	ZP	$Q_{0.05}$	$Q_{0.5}$	$Q_{0.95}$	ZMNB	ZINB	ZMP*	ZIP	NB	Poisson	N
1	59	777	1012	1315	1.00	1.00	1.00	1.00	1.00	1.00	906
2	59	74	102	138	1.00	1.00	1.00	1.00	1.00	1.00	839
3	29	769	1011	1334	1.00	1.00	1.00	1.00	1.00	1.00	1065
4	29	73	102	139	1.00	1.00	1.00	1.00	1.00	1.00	960
Scenario	ZP	$Q_{0.05}$	$Q_{0.5}$	$Q_{0.95}$	ZMNB	ZINB	ZMP	ZIP*	NB	Poisson	N
1	59	782	1015	1318	1.00	1.00	1.00	1.00	1.00	1.00	954
2	59	74	103	139	1.00	1.00	1.00	1.00	1.00	1.00	816
3	29	769	1015	1340	1.00	1.00	1.00	1.00	1.00	1.00	1015
4	28	73	102	139	1.00	1.00	1.00	1.00	1.00	1.00	936

3 R codes

3.1 rqr.glmmtmb

```
rqr.glmmtmb <- function(object)
{
  family = family(object)$family
  mu = predict(object,type="conditional")
  size = sigma(object)
  p = predict(object,type="zprob")
  n = object$modelInfo$nobs
  y = object$frame[,1]
  dzpois = function(x,lambda,p){
  return((1-p)*dpois(x,lambda)+p*(x==0))
  }
  pzpois = function(x,lambda,p){
  return((1-p)*ppois(x,lambda)+p*(x>=0))
  }
  dznbinom = function(x,size,mu,p){
  return((1-p)*dnbinom(x,size = size, mu = mu)+p*(x==0))
  }
  pznbinom = function(x,size,mu,p){
  return((1-p)*pnbinom(x,size = size, mu = mu)+p*(x>=0))
  }
  if(1*(object$modelInfo$allForm$ziformula==~0)==1)
  {
    if(family[1]=="gaussian")
    {
      pvalue = pnorm(y,mu,size)
    }
    else if(family[1]=="poisson")
    {
      pvalue = ppois(y-1,mu) + dpois(y,mu) * runif(n)
    }
    else if(family[1]=="nbinom2")
    {
      pvalue = pnbinom (y-1,size=size, mu=mu) + dnbinom (y,size = size, mu = mu)
    }
  }
  else if(1*(object$modelInfo$allForm$ziformula==~0)==0)
  {
    if(family[1]=="poisson")
    {
      pvalue = pzpois(y-1,mu,p) + dzpois(y, mu,p) * runif(n)
    }
    else if(family[1]=="nbinom2")
```

```
    {
      pvalue=pznbinom(y-1,size,mu,p) + dznbinom(y,size,mu,p) * runif(n)
    }
  }
  pvalue = pmin(pmax(pvalue,10^{-10}),1-10^{-10})
  qnorm2 = qnorm(pvalue)
  test = shapiro.test(qnorm2)$p.value
  list(pvalue=pvalue,qnorm2=qnorm2,test=test)
}
```

3.2 rqr.hurdle.glmmtmb

```
library(actuar)
rqr.hurdle.glmmtmb <- function(model_count, model_zero, data)
{
  name.y = names(model_count$frame)[[1]]
  y = data[,name.y]
  m = model_zero$modelInfo$nobs
  family = family(model_count)$family
  mu = rep(0, dim(data)[1])
  mu2 = predict(model_count,newdata=data[data[,name.y]>0,],type="conditional")
  mu[which(data[,name.y]>0)] = mu2
  size = sigma(model_count)
  prob_nb = size/(size+mu)
  pi= 1-predict(model_zero,newdata = data, type="zprob")
  if(family[1]=="truncated_poisson")
  {
    pvalue = pzmpois(y-1,mu,pi)+dzmpois(y,mu,pi)* runif(m)
  }
  else if(family[1]=="truncated_nbinom2")
  {
    pvalue = pzmnbinom(y-1,size,prob_nb,pi)+dzmnbinom(y,size,prob_nb,pi)*runif(m
  }
  pvalue = pmin(pmax(pvalue,10^{-10}),1-10^{-10})
  qnorm2 = qnorm(pvalue)
  test = shapiro.test(qnorm2)$p.value
  list(pvalue=pvalue,qnorm2=qnorm2,test=test)
}
```
